# Small Intestinal Bacterial Overgrowth and Pediatric Obesity—A Systematic Review

**DOI:** 10.3390/nu17091499

**Published:** 2025-04-29

**Authors:** Ana Maria Koller, Maria Oana Săsăran, Cristina Oana Mărginean

**Affiliations:** 1 Doctoral School, “George Emil Palade” University of Medicine, Pharmacy, Science, and Technology of Targu Mures, Gheorghe Marinescu Street No 38, 540136 Targu Mures, Romania; kolleranamaria@gmail.com; 2 Department of Pediatrics 3, “George Emil Palade” University of Medicine, Pharmacy, Science, and Technology of Targu Mures, Gheorghe Marinescu Street No 38, 540136 Targu Mures, Romania; 3 Department of Pediatrics 1, “George Emil Palade” University of Medicine, Pharmacy, Science, and Technology of Targu Mures, Gheorghe Marinescu Street No 38, 540136 Targu Mures, Romania; marginean.oana@gmail.com

**Keywords:** pediatric obesity, small intestinal bacterial overgrowth (SIBO), gut microbiota, systemic inflammation, metabolic dysfunction

## Abstract

**Background/Objectives:** Childhood obesity is a growing global concern linked to metabolic disorders such as nonalcoholic fatty liver disease (NAFLD). Small intestinal bacterial overgrowth (SIBO) may exacerbate these conditions by promoting systemic inflammation and metabolic dysfunction. This review evaluates the prevalence of SIBO in obese children, its association with inflammatory and metabolic markers, and the efficacy of diagnostic and therapeutic strategies. **Methods:** A systematic search of PubMed, Scopus, and Web of Science (2010–present) was conducted using Boolean operators: (‘small intestinal bacterial overgrowth’ OR ‘SIBO’) AND ‘prevalence’ AND (‘low-grade inflammatory markers’ OR ‘metabolic status’) AND ‘gut microbiome’ AND ‘dysbiosis’ AND ‘obese children’. **Results:** The data show that SIBO is frequently observed in obese pediatric populations and is associated with gut dysbiosis, impaired nutrient absorption, and reduced production of short-chain fatty acids. These changes contribute to increased intestinal permeability, endotoxemia, and chronic low-grade inflammation. Several microbial taxa have been proposed as biomarkers and therapeutic targets. Diagnostic inconsistencies persist, but treatments such as probiotics, prebiotics, dietary interventions, and selective antibiotics show potential, pending further validation. **Conclusions:** Early identification and treatment of SIBO with tailored strategies may help reduce metabolic complications and improve outcomes in children with obesity.

## 1. Introduction

Childhood obesity is a major global health issue, particularly in high-income countries, and is strongly associated with long-term complications such as type 2 diabetes, cardiovascular disease, and certain cancers [1,2,3]. Over the past three decades, obesity rates have surged, contributing to more than 3 million deaths annually worldwide [4,5,6]. The underlying causes are multifactorial, but a consistent driver is the imbalance between caloric intake and expenditure, which also promotes chronic low-grade inflammation [7].

In recent years, attention has turned to the role of the gut microbiota in obesity-related metabolic dysfunction. Among the microbial imbalances observed, Small Intestinal Bacterial Overgrowth (SIBO), defined as an excessive proliferation of bacteria in the upper gastrointestinal tract, has gained increasing interest [8]. While traditionally associated with undernutrition and malabsorption [9], newer evidence suggests that SIBO also affects individuals with obesity, possibly contributing to systemic inflammation and metabolic disturbances [10,11,12]. Proposed mechanisms include immune dysregulation and bacterial translocation to metabolically active tissues, which may amplify inflammatory responses and disrupt metabolic homeostasis [13,14,15].

Comorbidities such as insulin resistance, dyslipidemia, hypertension, and nonalcoholic fatty liver disease (NAFLD) are common in obese children and are often fueled by persistent inflammation. Elevated markers such as C-reactive protein (CRP) and interleukin-6 (IL-6) are frequently observed, reflecting the inflammatory burden in this population [16,17,18,19]. Gut microbiota composition, including the presence of SIBO, has been implicated in mediating these effects [20].

Although various therapeutic strategies have been proposed, including proton pump inhibitors (PPIs), antibiotics, probiotics, and prebiotics, their effectiveness and safety profiles differ. PPIs, while useful for acid suppression, may increase SIBO risk by altering the gastric environment [21,22,23]. In contrast, probiotics and prebiotics aim to restore microbial balance and may reduce inflammation, though evidence of their long-term efficacy remains limited [24]. Antibiotics, which are effective in eradicating bacterial overgrowth, must be used cautiously to avoid exacerbating dysbiosis [25,26].

Given the growing evidence linking SIBO with pediatric obesity and its metabolic complications, a clearer understanding of this relationship is essential. This review *aims* to synthesize current findings on the prevalence of SIBO in obese children, examine its association with systemic inflammation and metabolic dysfunction, and evaluate available diagnostic tools and therapeutic interventions.

## 2. Materials and Methods

A.M.K. and M.O.S., supervised by C.O.M., conducted a comprehensive search of Web of Science, Scopus, and PubMed for studies published from January 2010 onward, examining the link between SIBO and obesity in children. The review also explored how low-grade inflammation and metabolic status influence gut microbiota in obese youth. Search terms included combinations of “SIBO”, “prevalence”, “low-grade inflammatory markers”, “metabolic status”, “gut microbiome”, “dysbiosis”, and “obese children”.

Only English full-text randomized controlled trials, cohort, cross-sectional, or longitudinal studies were included. Case studies, reviews, meta-analyses, non-English papers, and duplicates were excluded in order to focus on primary evidence. The details regarding the search strategy applied, and the types of articles included are represented in Table 1.

In this systematic review, all 24 included studies were assessed for methodological quality using the NIH Study Quality Assessment Tools [27]. Each tool was applied in full according to the study design (e.g., observational, cross-sectional, or RCT), and responses to individual items were recorded as “Yes”, “No”, “NR” (Not Reported), “NA” (Not Applicable), or “CD” (Cannot Determine). Studies with 11 or more “Yes” responses (out of 13–14 items, depending on the checklist used) and no major methodological concerns were classified as having a low risk of bias. Those with 7–10 “Yes” responses and 3–6 “No” or “NR” responses, often due to small sample size, retrospective design, or insufficient control for confounding, were rated as moderate risk. No studies were rated as high risk of bias. This classification allowed us to systematically interpret the robustness of findings across diverse study types (Table 2).

## 3. Results

**Figure 1:**
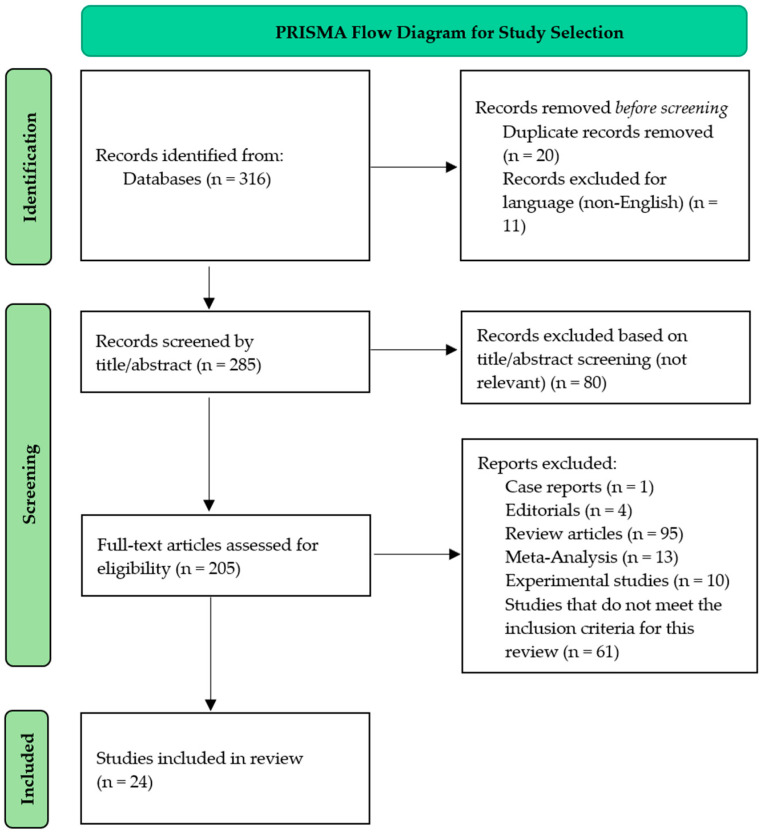
PRISMA flowchart for assessment of eligible studies.

The search yielded 316 results, which were reduced to 285 after deleting duplicates and non-English language entries. Figure 1 depicts the complete article selection procedure as outlined in the PRISMA 2020 guidelines and revised flow diagrams. Eighty items were removed because they were irrelevant to the topic matter. After excluding experimental research, meta-analyses, review articles, one case report, and four editorials, a total of 24 publications remained. These researches primarily sought to determine the incidence of SIBO in overweight and obese children, to investigate the link between SIBO and systemic inflammation in this group, and to examine the impact of different therapies on the gut microbiota (Table 3).

### 3.1. Correlation Between SIBO and Obesity in Pediatric Populations

Emerging evidence highlights a consistent link between pediatric obesity and gut microbiota composition. Compared to lean peers, obese children exhibit distinct microbial signatures characterized by increased levels of *Lactobacillus*, *Prevotella*, *Escherichia coli*, and *Clostridium*, alongside decreased abundances of Bifidobacterium and Bacteroides species [40,50,51]. These alterations are associated with systemic inflammation, altered nutrient absorption, and greater energy harvest from the diet.

Diet plays a central role in shaping gut microbial profiles. High-fiber, plant-based diets are linked with elevated *Bacteroidetes* (e.g., *Prevotella*), while Western, high-fat diets favor Firmicutes and pro-inflammatory taxa [40,52]. Obesity is also often associated with reduced microbial diversity and an increase in energy-efficient bacteria such as *Faecalibacterium*, *Roseburia*, and *Lachnospiraceae*, which produce short-chain fatty acids like butyrate. Paradoxically, despite this enrichment, obese children may exhibit reduced butyrate levels, indicating functional dysbiosis [3,39,53,54,55,56].

Sex-based differences further shape the gut microbiota, particularly during puberty. In pediatric populations, girls with obesity tend to exhibit a greater diversity of *Lactobacillus* and *Bifidobacterium* species, whereas boys more frequently harbor *Bilophila* and *Phascolarctobacterium* species. These microbial patterns appear to be influenced by pubertal hormonal shifts, as estrogen and androgens modulate bacterial growth and metabolism, a dynamic increasingly studied within the framework of the emerging “microgenderome” concept [51,57,58,59,60,61,62,63,64] (Table 4).

At the genus level, Bacteroides is often inversely associated with BMI and inflammation, while *Prevotella* shows a positive correlation with the same parameters [3,65,66]. While inter-study variability exists, current findings collectively suggest that gut dysbiosis plays a pivotal role in the metabolic dysfunctions seen in pediatric obesity.

### 3.2. The Factors Involved in Offspring’ Gut Microbiota

Maternal obesity affects the gut flora of infants, increasing their risk of obesity through vertical transmission mechanisms such as vaginal delivery and breastfeeding [54,67]. Infants born to obese mothers often harbor increased levels of *Parabacteroides* and *Oscillibacter*, microbial genera associated with metabolic dysregulation and obesity, likely transmitted from the maternal gut [55,67]. These microbial shifts influence immunological and metabolic responses, thereby elevating the risk of obesity-related diseases later in life [39].

Along with maternal implications, birth style shapes newborn gut flora. Mothers’ vaginal and intestinal flora consists mostly of *Lactobacillus*, *Sneathia*, and *Prevotella* [68]. With less diversity and lower amounts of *Bacteroides* and *Bifidobacteria*, the microbiota left behind after cesarean birth mimics that of the mother’s skin. This change increases young children’s likelihood of obesity [69].

Microbial spread is largely influenced by the mother’s nutrition and lifestyle. While mother obesity lowers *Bifidobacterium* and increases *Staphylococcus* levels in breast milk, a high-fat diet reduces *Bacteroidetes* in newborns, therefore impacting microbial development [70]. Shaped by maternal health and delivery techniques, the early-life microbiome may have long-term metabolic consequences [71].

*Actinobacteria* decrease during this period, while *Firmicutes*, *Bacteroidetes*, and *Proteobacteria*, all of which are vital for the microbiome, increase [72]. The variability is influenced by geography, antibiotic exposure, and lactation [71]. Reduced microbial diversity resulting from prenatal antibiotics has a long-term impact on metabolism [31].

### 3.3. NAFLD in Children—Risks and Prevention

NAFLD, affecting both obese and overweight children, represents a leading cause of chronic liver disease in pediatric populations. Usually asymptomatic, it is underdiagnosed in children [73]. Comparable to the U.S. prevalence of 17% and the Australian teenage prevalence of 10%, the Australian Raine Pregnancy Cohort noted a 13% prevalence at 17 years [74]. Metabolic changes associated with puberty may influence the development of NAFLD in children through mechanisms distinct from those observed in adults [75]. Importantly, obesity is not merely a risk factor but a direct causal contributor to NAFLD through mechanisms that include increased visceral adiposity, insulin resistance, and chronic low-grade inflammation. High fructose intake facilitates hepatic fat accumulation and metabolic dysfunction, thereby increasing the risk of developing NAFLD [42,76,77] (Table 5). Accordingly, reducing the intake of foods with added sugar consumption may help improve hepatic steatosis and metabolic parameters such as insulin sensitivity and liver enzyme levels in obese adolescents diagnosed with NAFLD [78].

As small intestinal bacterial overgrowth (SIBO) emerges as a significant driver of metabolic dysregulation in obesity, the gut microbiota has become increasingly recognized as a central regulator of both metabolic homeostasis and liver function [79]. SIBO is characterized by excessive bacterial colonization in the small intestine, which, in turn, increases intestinal permeability and allows bacterial products such as lipopolysaccharides (LPS) to enter the systemic circulation. Considered “metabolic endotoxemia”, this disorder can lead to hepatic steatosis, insulin resistance, and systemic inflammation [80]. Obesity induces alterations in gut microbiota composition, further exacerbating SIBO and its associated metabolic consequences [81].

The prevalence of SIBO is significantly higher among patients with nonalcoholic steatohepatitis (NASH), ranging from 50% to 78%, compared to non-obese individuals [79]. Studies have indicated that SIBO causes hepatic inflammation by activating toll-like receptor 4 (TLR4) and generating cytokines such as tumor necrosis factor-alpha (TNF-α) and interleukin 8 (IL-8) [43,82] (Table 5). This cascade not only contributes to NAFLD but also promotes the development of hepatic fibrosis [81,83]. Furthermore, another mechanism connected to SIBO is the endogenous ethanol generation by gut bacteria, which contributes to liver mitochondrial dysfunction and oxidative stress [84]. These mechanisms highlight the central role of SIBO within the gut-liver axis and its role in NAFLD pathogenesis in obese young patients [85].

SIBO and NAFLD interact, revealing an intricate connection mediated by the gut-liver axis. SIBO-related enhanced intestinal permeability allows bacterial products such as LPS to be translocated into the portal circulation [86]. Once in the liver, these products rely on TLR4 signaling to activate Kupffer cells and hepatic stellate cells, hence generating inflammatory and profibrotic reactions [87]. Furthermore, the dysbiosis associated with SIBO alters the intestinal metabolism of dietary substrates, leading to the depletion of essential nutrients such as choline and the production of metabolites like trimethylamine-N-oxide (TMAO), which disrupt lipid metabolism and contribute to hepatic fat accumulation [88].

Pediatric research indicates that obese children with SIBO are significantly more likely to develop NAFLD compared to those without dysbiosis [89]. This association is not only linked to metabolic issues like insulin resistance and dyslipidemia but also to elevated liver enzymes, such as alanine aminotransferase (ALT) and aspartate aminotransferase (AST) [35]. These findings underscore the importance of early identification and treatment of SIBO as part of a comprehensive strategy for managing NAFLD in obese children [29].

Targeting SIBO and restoring gut microbial balance represent promising therapeutic avenues for reducing NAFLD in obese pediatric populations [90]. In terms of changing gut microbiota composition, decreasing intestinal permeability, and therefore relieving hepatic inflammation, probiotic and prebiotic treatment has shown success [91]. In both pediatric and adult NAFLD patients, clinical trials including species of *Lactobacillus* and *Bifidobacterium* have shown increases in liver enzyme levels, decreases in hepatic fat content, as well as improved insulin sensitivity [92,93,94].

Similarly, dietary adjustments aimed at reducing fructose and refined carbohydrate intake are beneficial for limiting de novo lipogenesis and improving hepatic fatty acid oxidation [46,95] (Table 5). These treatments may substantially reduce hepatic fat and enhance liver function in obese children when combined with weight loss programs and physical activity [96].

**Table 5:** SIBO and liver health in NAFLD and obesity.

Study (Year)	Study Type	Population	Diagnostic Tool (s)	Main Outcome (s)
Belei et al., 2017 [29]	2-year prospective case-control study	125 overweight/obese children (10–18 years) and 120 healthy controls	GHBT (SIBO), abdominal ultrasound and liver enzymes (NAFLD), metabolic syndrome parameters	SIBO prevalence was 37.6% in obese children vs. 3.3% in controls. SIBO-positive obese children had a significantly higher NAFLD prevalence (59.5% vs. 10.2%) and more frequent elevated ALT/AST, hypertension, and metabolic syndrome.
Browning et al., 2011 [46]	Randomized dietary intervention (2-week trial)	18 adults with obesity and NAFLD (mean age 45; BMI ~35)	MRI spectroscopy (hepatic fat content)	Both low-carb and low-calorie diets reduced liver fat (~42%), but a low-carb diet led to a greater reduction (−55% vs. −28%; *p* = 0.008). AST decreased with weight loss; ALT remained unchanged.
Abdelmalek et al., 2010 [42]	Multicenter cross-sectional observational study	427 adults with biopsy-confirmed NAFLD	Food-frequency questionnaire (fructose intake); liver histology (steatosis, inflammation, fibrosis); metabolic syndrome parameters	Daily fructose intake was linked to younger age, higher BMI and triglycerides, lower steatosis grade, but more advanced fibrosis. Daily consumers had ~2.6× higher odds of advanced fibrosis (*p* = 0.004).
Guercio Nuzio et al., 2017 [35]	Cross-sectional case-control study	23 obese children (mean age 11 years) and 9 healthy controls	Lactulose: mannitol test (intestinal permeability), LHBT (SIBO), serum endotoxin, blood ethanol, fecal calprotectin, ultrasound and ALT (steatosis)	48% of obese children had increased intestinal permeability. SIBO was only detected in the obese group. Elevated permeability was associated with higher endotoxin (r = 0.48) and ethanol and was a risk factor for steatosis (*p* < 0.002).
Shanab et al., 2010 [94]	Comparative case-control study	18 adults with biopsy-confirmed NASH and 16 age- and sex-matched healthy controls	LHBT (SIBO), LBP (endotoxemia), TLR-4 (monocytes), plasma cytokines (IL-1β, IL-6, IL-8, TNF-α)	SIBO prevalence was higher in NASH (77.8%) vs. controls (31.3%). Only IL-8 was significantly elevated (*p* = 0.04) and correlated with TLR-4 expression (r = 0.51). No differences in LBP or other cytokines.

ALT—Alanine Aminotransferase; AST—Aspartate Aminotransferase; BMI—Body Mass Index; GHBT—Glucose Hydrogen Breath Test; IL—Interleukin; LBP—Lipopolysaccharide-Binding Protein; LHBT—Lactulose Hydrogen Breath Test; MRI—Magnetic Resonance Imaging; NAFLD—Nonalcoholic Fatty Liver Disease; NASH—Nonalcoholic Steatohepatitis; SIBO—Small Intestinal Bacterial Overgrowth; TLR-4—Toll-Like Receptor 4; TNF-α—Tumor Necrosis Factor Alpha.

### 3.4. Impact of Proton Pump Inhibitors, Probiotics, Prebiotics, and Antibiotics on Obese Children with SIBO

#### 3.4.1. The Role of PPIs in Pediatric Obesity and SIBO

PPIs represent the first-line treatment for gastroesophageal reflux disease (GERD) in both children older than one year and adults, typically involving a 4-week course of either histamine H2 receptor antagonists or PPIs [97]. Despite their proven efficacy, PPIs alter the intraluminal environment and gut microbiota, thereby contributing to the development of SIBO, with hypochlorhydria playing a key role [98,99] (Table 6).

The widespread use of over-the-counter PPIs in pediatric populations, combined with their perceived safety, underscores the need to evaluate potential complications such as SIBO [100]. Belei et al. observed that more than 60% of children with GERD treated with PPIs had symptoms related to SIBO [30]. Similarly, the cited study supports Sieczkowska et al.’s conclusion that children undergoing PPI treatment should be screened for SIBO [28] (Table 6).

Rosen et al. reported that acid suppression may favor the overgrowth of specific bacterial strains, such as *Staphylococcus* and *Streptococcus*, resulting in stomach bacterial overgrowth. Moreover, their findings indicated that non-acid reflux was linked to increased bacterial colonization in the lungs, increasing the risk of respiratory tract infections [101].

According to Liang et al. [102], more than half of patients with gastrointestinal cancer had been taking PPIs for a long time, and more than 70% of these patients tested positive for SIBO. They found that only 19% of patients who received probiotic supplementation (Bifidobacterium) alongside PPIs tested positive for SIBO [102].

Belei et al. further reported that approximately 30% of children with GERD who had been using PPIs for three months tested positive for SIBO, with probiotics significantly reducing this rate [30] (Table 6). Similarly, Lombardo et al. found a substantial connection between PPI usage and an increased risk of SIBO, as evaluated by a hydrogen-glucose breath test (GHBT) [103]. Furthermore, Choung et al. found microbiome changes in adult PPI users, corroborating this link [104].

In conclusion, probiotic supplementation, particularly with *Lactobacillus reuteri*, has been shown to reduce the incidence of SIBO and its associated symptoms in children taking PPIs, emphasizing the importance of regular probiotic administration in managing pediatric GERD to prevent SIBO [30] (Table 6).

#### 3.4.2. Probiotics as a Mitigating Strategy for PPI-Induced SIBO

Histamine H2 receptor antagonists and PPIs are commonly administered to premature neonates to prevent gastrointestinal bleeding and manage gastroesophageal reflux. However, suppression of gastric acid impairs the stomach’s natural defense mechanisms, increasing the risk of systemic infections and necrotizing enterocolitis (NEC) [105].

Very low birth weight (VLBW) infants exposed to these medications are at an increased risk of developing infections, particularly from *Gram-negative bacilli* and *Candida*. The risk increases proportionally with the duration of exposure [106]. Moreover, *Candida* colonization, an important risk factor for systemic infections, is more frequent in preterm neonates due to their underdeveloped immune systems [107].

Prolonged exposure to proton pump inhibitors proportionally increases the risk of *Candida* colonization and systemic infection, with each day of therapy contributing to a higher cumulative risk [32,106]. However, bovine lactoferrin (BLF), as well as its combination with *Lactobacillus rhamnosus GG* (LGG), has been shown to inhibit microbial colonization, support intestinal barrier maturation, and lower the incidence of systemic infections [32,107].

For VLBW newborns, a gradual reduction of PPI dosage is recommended following the initial day of treatment [32,108]. BLF has been shown to reduce infection rates by more than half while also lowering the risk of NEC and enhancing both digestive and immune system protection [109].

#### 3.4.3. Antibiotics and Their Impact on Gut Dysbiosis in SIBO Management

Although essential in the treatment of SIBO, antibiotics can disrupt the gut microbial equilibrium, often resulting in dysbiosis [110]. Their use increases the risk of *Clostridium difficile* infection (CDI), particularly when agents such as clindamycin, fluoroquinolones, or cephalosporins are administered [111]. Broad-spectrum antibiotics, such as vancomycin and amoxicillin, inhibit beneficial *Firmicutes* and reduce secondary bile acid secretion, creating an environment that favors *C. difficile* colonization [112]. A 30-day course of antibiotics has been associated with a twelvefold increase in the risk of CDI [113].

Clindamycin has been shown to eliminate up to 90% of cecal bacterial taxa, with its effects persisting for up to ten days, which typically recovers within four weeks [111,114]. Furthermore, *C. difficile* infection can hinder the recovery of beneficial bacteria through competitive interactions [111].

#### 3.4.4. The Role of Prebiotics in Restoring Gut Microbial Balance

Multiple factors influence the composition and stability of the gut microbiota, including genetics, food, antibiotic exposure, and chronic inflammatory gastrointestinal disorders [45,115]. The primary contributing factors to SIBO include gastric hypochlorhydria and impaired small intestinal motility [116]. According to recent research, while the frequency of SIBO does not vary much between PPIs, pantoprazole has a statistically significant connection with the development of this disease [103,117].

Prokinetics reduces the risk of SIBO by increasing gastrointestinal contractility and encouraging the effective passage of intestinal contents [118]. Patients receiving a combined regimen of PPIs and prokinetics, such as domperidone or levosulpiride, have a considerably lower prevalence of SIBO (only 3%) than those who simply receive PPIs [45] (Table 6).

Furthermore, these therapeutic combinations have been shown to reduce intestinal transit time, further supporting the role of prokinetics in restoring physiologic intestinal motility [119]. Prolonged PPI use, especially beyond six months, significantly increases the risk of developing SIBO [103]. Nevertheless, prolonged use of prokinetic agents has been associated with adverse effects, including extrapyramidal symptoms, hyperprolactinemia, and cardiac arrhythmias [45] (Table 6).

### 3.5. SIBO Diagnosis—Methods and Pediatric Considerations

The GHBT is the most widely used diagnostic method for SIBO and must be preceded by a fasting period [120]. After ingesting a glucose solution (2 g/kg, max 50 g), patients undergo hydrogen level measurements every 15 min over a time span of two hours. An increase of more than 12 ppm above baseline, or a peak value exceeding 20 ppm, is considered diagnostic for SIBO [121]. Adequate pre-test preparation is critical, as individuals with SIBO frequently exhibit elevated baseline hydrogen concentrations [122].

The prevalence of SIBO varies significantly across populations. For example, Korterink et al. reported a 14.3% prevalence in Dutch children with functional gastrointestinal disorders, predominantly irritable bowel syndrome (IBS) [37]. The Lactulose Hydrogen Breath Test (LHBT), although less specific, is more appropriate for identifying abnormal orocecal transit patterns since methane can slow motility and hydrogen fermentation may lead to diarrhea [123,124,125]. Certain microbes, such as *Veillonella*, have been linked to IBS and SIBO-related symptoms [126].

Breath testing typically involves the measurement of hydrogen (H_2_) and methane (CH_4_), with diagnostic thresholds defined as H_2_ > 20 ppm or CH_4_ ≥ 10 ppm within 60 min [127]. Elevated breath methane levels appear to be more prevalent among children from socioeconomically disadvantaged settings, a factor that may contribute to the increased overall prevalence of positive methane findings in diagnostic breath testing [128,129]. Simultaneous measurement of both gases enhances diagnostic accuracy, though not all IBS patients show increased levels [123,128,130].

Common non-invasive tests include GHBT, LHBT, and the carbon-14-labeled D-xylose breath test (XBT), which demonstrates a sensitivity/specificity range of 60–100% [123,131,132]. Results may be affected by factors such as vomiting, dehydration, prior abdominal surgery, or abnormal gastrointestinal motility [133].

SIBO correlates with low BMI, corticosteroid use, and surgeries such as ileocecal resections and cholecystectomies [132,134,135,136,137,138,139]. Additional risk factors include female sex, advanced age, GI disorders, and immunosuppressive therapies [132].

In summary, GHBT is the preferred method for detecting proximal SIBO, whereas LHBT remains a valuable alternative when distal overgrowth is suspected or when GHBT is unavailable [123,140] (Table 7).

## 4. Discussion

Recent studies investigating the association between SIBO and pediatric obesity have identified notable correlations [9]. However, these findings require cautious interpretation due to diagnostic inconsistencies and methodological constraints [41,73]. While many studies report a high prevalence of SIBO in overweight or obese children, causality remains unproven and potentially confounded by factors such as diet, gastrointestinal motility, medication use, and socioeconomic context [10,11,12].

### 4.1. Interpreting Diagnostic Complexities

Breath testing, the most common non-invasive diagnostic method for SIBO, presents notable challenges [130,141,142]. The glucose hydrogen breath test (GHBT) offers higher specificity by targeting the proximal small intestine, thereby minimizing false positive test results due to colonic fermentation [37,121,123]. However, it may fail to detect distal SIBO [123]. Conversely, the LHBT allows for assessment of the entire intestinal tract but is prone to false positives due to rapid orocecal transit [123,124,143]. Yu et al. [124] demonstrated that in 88% of IBS patients with an early rise in breath hydrogen during LHBT, the test meal had already reached the cecum. This suggests that the hydrogen increase was the result of colonic fermentation rather than small intestinal bacterial overgrowth. Therefore, a positive LHBT may primarily reflect orocecal transit rather than true bacterial overgrowth [124].

In pediatric populations, GHBT has been used with thresholds such as a hydrogen rise ≥20 ppm or ≥12 ppm above baseline, with SIBO prevalence reported between 14.3% and 31% [37,121]. Nevertheless, these studies often lack control for fast transit or confounding factors, reducing diagnostic certainty [123,124,140]. Although the 14C-D-xylose breath test (XBT) has shown promise in adults, it is rarely used in children and may be affected by hepatic or renal dysfunction [131].

### 4.2. Disparities in Prevalence Reports

Moreover, reported SIBO prevalence differs markedly across populations, from 3.3% in healthy controls to over 70% in high-risk subgroups [29,37,41,94]. This likely reflects differences in diagnostic tools, study populations, and definitions [26,123]. Few studies employ objective transit assessments or standardized testing criteria, thereby making cross-study comparisons difficult.

### 4.3. Evaluating Inflammation and Metabolic Consequences

Furthermore, associations between SIBO and elevated inflammatory markers such as CRP, IL-6, and TNF-α have been inconsistently reported [3]. For instance, Esposito et al. found higher cytokine levels in SIBO-positive obese children, though without correlation to SIBO severity [41]. Such discrepancies underscore the importance of controlling for infection, diet, and medication use when interpreting these markers [3,66,144,145,146].

### 4.4. Microbiota Profiles in the Context of Obesity

Equally relevant, multiple studies have characterized the gut microbial composition in obese versus lean children. A consistent pattern emerges: increased *Prevotella* and *Lactobacillus* and decreased *Bacteroides* and *Bifidobacterium* in obese children [50]. Yet, studies differ in detail. For example, Hu et al. reported lower *Bacteroides* and higher *Prevotella* levels in obese adolescents [3], while Ignacio et al. found higher levels of *Bacteroides* fragilis in obese children [40]. These contradictions may reflect methodological differences, dietary influences, or regional variation [5,55,147].

Paradoxically, elevated levels of *Faecalibacterium*, typically considered anti-inflammatory, have been observed in some obese cohorts, raising questions about its functional role [39,54,55]. The *Firmicutes/Bacteroidetes* ratio also shows an inconsistent association with obesity, possibly due to its dynamic nature across developmental stages [3,148,149].

Gender-specific microbiota profiles have also been described. For example, obese females often exhibit more diverse *Lactobacillus* and *Bifidobacterium* species, potentially due to estrogen-mediated effects [58,59,64]. These differences suggest that sex hormones influence gut microbial development, particularly during puberty [63].

### 4.5. Implications for Management and Treatment

In terms of clinical management, PPIs are frequently used in children with GERD but have been linked to increased SIBO risk by reducing gastric acidity [30,97,105]. For example, Belei et al. reported a SIBO prevalence of 56.2% in PPI-treated children, which fell to 6.2% with concurrent probiotic (*L. reuteri*) administration [30]. These results support a potential protective role for probiotic co-administration during acid suppression therapy.

Antibiotics, while effective for eradicating SIBO, may cause dysbiosis and increase *Clostridium difficile* risk [110,111]. Conversely, probiotics and prebiotics offer safer alternatives for restoring microbial balance, although their long-term efficacy remains unclear [30,45,102,112].

In addition, prebiotic and synbiotic therapies have shown improvements in liver function and inflammatory profiles in children with obesity-related NAFLD, potentially by reducing intestinal permeability and endotoxemia [47,48,49,79,86]. These approaches warrant further testing in longitudinal studies.

The current body of evidence is limited by small sample sizes and a moderate risk of bias in many of the included studies, primarily due to inadequate control of confounders such as diet, medication use, and socioeconomic factors. Most studies were cross-sectional, further limiting causal inference. Variability in breath test methods and diagnostic thresholds further complicates interpretation and reduces generalizability.

### 4.6. Persistent Limitations and Areas for Advancement

Despite these insights, most included studies were cross-sectional with a moderate risk of bias due to small samples, lack of blinding, and confounding variables. Even among RCTs, variability in intervention protocols and outcome definitions complicates interpretation. Furthermore, populations studied varied widely in age, diet, and comorbidities, limiting generalizability [55,72,150]. Socioeconomic disparities, noted in studies from Brazil and Mexico, also likely influence both SIBO prevalence and gut microbiota composition [7,128].

Inconsistent diagnostic approaches contribute significantly to heterogeneity [26]. GHBT and LHBT differ in sensitivity and specificity, and their interpretation is often unstandardized [123]. Adjunctive assessments of gut transit and validated biomarkers are rarely used, thus limiting confidence in prevalence estimates [119,124,132].

Clarifying the role of SIBO in pediatric obesity will require future research to:

- Standardize SIBO diagnostic criteria and testing protocols
- Include longitudinal, multicenter cohorts to establish causality
- Control for confounders such as diet, medication, and socioeconomic status
- Investigate microbiota-based biomarkers for early detection and therapeutic targeting

**Figure 2:**
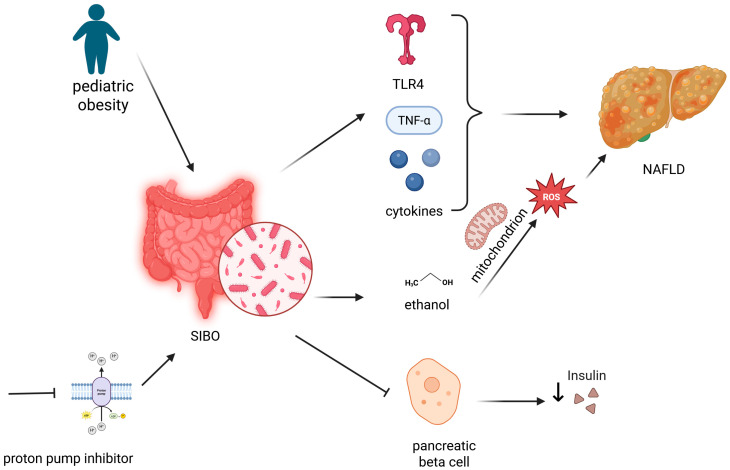
Interplay between SIBO, inflammation, NAFLD and pediatric obesity. (created with https://app.biorender.com/). Legend: Pediatric obesity and proton pump inhibitor use promote the development of SIBO in children. The subsequent promotion of cytokine release, including TLR4 and TNF-α, stimulates the development of NAFLD, together with the production of ethanol by bacterial overgrowth, which stimulates the release of ROS by the mitochondrion. Nevertheless, SIBO determines a decrease in insulin production through impairment of pancreatic beta cell function. NAFLD—nonalcoholic fatty liver disease; SIBO—small intestinal bacterial overgrowth; TLR4—toll-like receptor 4; TNF-α—tumor necrosis factor-alpha.

Altogether, a conceptual model is emerging where obesity-associated dysbiosis promotes SIBO, which in turn increases intestinal permeability and endotoxin translocation, thereby amplifying systemic inflammation and metabolic dysfunction [82,93,151]. This gut-liver axis may be especially relevant in pediatric NAFLD, where SIBO is prevalent and contributes to disease progression [35,89,92]. A schematic representation of the interplay between SIBO, obesity, inflammation and the development of NAFLD has been provided in Figure 2.

In conclusion, while current evidence indicates an associative relationship between SIBO and pediatric metabolic dysfunction, methodological refinement is essential before SIBO can be considered a modifiable therapeutic target. With rigorous future research, microbiota-based strategies may eventually complement lifestyle and pharmacologic approaches in managing obesity-related complications in children.

Standardizing SIBO diagnosis in children is essential. We recommend using the GHBT with a ≥12 ppm rise or ≥20 ppm peak within 90 min. Testing should include both hydrogen and methane where possible and follow uniform pretest prep. In unclear cases, LHBT may complement GHBT. Establishing expert consensus on protocols would improve research consistency.

## 5. Conclusions

Although SIBO appears to be more prevalent among overweight and obese children, current evidence supports an associative rather than a causal relationship. Evidence for its potential role in promoting intestinal permeability, inflammation, and metabolic dysfunction is constrained by inconsistent diagnostic methods, variable study quality, and population heterogeneity. The lack of standardized breath testing protocols and insufficient adjustment for confounding factors such as diet and medication use further complicate data interpretation.

Thus, while early detection and individualized treatment of SIBO may hold clinical promise, such approaches should be pursued with caution. Clarifying the role of SIBO in pediatric obesity requires the implementation of standardized diagnostic protocols, longitudinal multicenter studies, and rigorously controlled interventions. Investigating microbiota-based biomarkers could facilitate early identification and the development of targeted interventions for high-risk pediatric populations.

Future research efforts must transition from observational associations to interventional studies in order to define when, how, and in which pediatric subgroups SIBO treatment can meaningfully impact the course of obesity-related metabolic disease.

## Figures and Tables

**Table 1:** Literature Search Strategy Overview.

Component	Details
Keywords	“small intestinal bacterial overgrowth” OR “SIBO” AND “prevalence” AND “low-grade inflammatory markers” OR “metabolic status” AND “gut microbiome” AND “dysbiosis” AND “obese children”
Databases Searched	PubMed, Scopus, Web of Science
Database-Specific Search Details	PubMed: (“small intestinal bacterial overgrowth” OR “SIBO”) AND “prevalence” AND “gut microbiome” AND “obese children” Scopus: (“SIBO” OR “small intestinal bacterial overgrowth”) AND “low-grade inflammatory markers” AND “metabolic status” Web of Science: (“small intestinal bacterial overgrowth” OR “SIBO”) AND “obesity” AND “children” AND “gut microbiota”
Timeframe	January 2010–Present
Language	English only
Study Types Included	Randomized controlled trials, cohort studies, cross-sectional studies, longitudinal studies
Additional Sources	References cited in included articles and selected reviews
Inclusion Criteria	- Full text in English - Investigating SIBO and obesity in pediatric populations - Data on gut microbiota, metabolic/inflammatory markers, or interventions
Exclusion Criteria	- Case reports - Editorials - Reviews - Meta-analyses - Non-English publications - Abstract-only articles - Duplicate entries
Study Categories	- Prevalence studies - Diagnostic methods - Therapeutic interventions (e.g., antibiotics, probiotics) - Microbiota and metabolic correlations

SIBO—Small Intestinal Bacterial Overgrowth.

**Table 2:** Risk of Bias Assessment of Included Studies (Based on NIH Quality Assessment Tool).

Author (Year)	Study Design	Risk of Bias	Justification
Sieczkowska et al. (2015) [28]	Prospective cohort	Moderate	Small sample size; limited confounder control; no blinding.
Belei et al. (2017) [29]	Cross-sectional	Moderate	Good comparison groups but observational; potential residual confounding.
Belei et al. (2018) [30]	RCT	Low	Randomization, placebo group, and outcome measurement are well-described.
Tapiainen et al. (2019) [31]	Prospective controlled cohort	Low	A well-designed prospective study with exposure/outcome measures clearly defined.
Manzoni et al. (2018) [32]	Secondary analysis of RCT	Moderate	Non-randomized comparison between exposure groups; secondary analysis.
Scott et al. (2016) [33]	Retrospective cohort	Moderate	Large sample but risk of misclassification bias from administrative data.
Todendi et al. (2016) [34]	Cross-sectional	Moderate	No causal inference; confounding variables are only partially controlled.
Murugesan et al. (2015) [7]	Cross-sectional	Moderate	Descriptive study with compositional analysis; limited generalizability.
Guercio Nuzio et al. (2017) [35]	Cross-sectional	Moderate	Small sample size; associations only.
Lai et al. (2019) [36]	RCT	Low	Placebo-controlled RCT with microbiota and clinical outcomes measured.
Korterink et al. (2015) [37]	Cross-sectional	Moderate	Adequate outcome measurement; confounding and selection bias likely.
Galley et al. (2014) [38]	Cross-sectional	Moderate	Microbiome analysis is valid but limited in controlling for SES and other variables.
McCann et al. (2018) [39]	Cross-sectional	Moderate	Small sample size; descriptive viral diversity analysis.
Ignacio et al. (2016) [40]	Cross-sectional	Moderate	Limited sample; single center; descriptive analysis.
Esposito et al. (2020) [41]	Cross-sectional	Moderate	Well-defined population, but no causal conclusions.
Hu et al. (2015) [3]	Cross-sectional	Moderate	Associative findings without adjustment for all relevant confounders.
Abdelmalek et al. (2010) [42]	Cross-sectional	Moderate	Liver biopsy adds rigor; dietary self-report introduces potential bias.
Abu-Shanab et al. (2011) [43]	Case-control	Moderate	Matched controls; potential recall bias; small sample.
Boursier et al. (2016) [44]	Cross-sectional	Moderate	Includes histological confirmation; possible confounding bias.
Revaiah et al. (2018) [45]	Cross-sectional	Moderate	Good comparison; non-randomized groups; confounders not fully adjusted.
Browning et al. (2011) [46]	RCT	Low	Controlled intervention with biological outcomes; small sample size noted.
Van Rossum et al. (2024) [47]	RCT	Low	Large multicenter RCT with microbiome and clinical endpoints.
Atazadegan et al. (2023) [48]	RCT	Low	Double-blinded RCT with objective outcomes.
Rahkola et al. (2023) [49]	RCT	Low	Randomized, placebo-controlled microbiota outcomes were measured; a small sample was acknowledged.

RCT—Randomized Controlled Trial; SES—Socioeconomic Status.

**Table 3:** Summary of included studies.

Author (Year)	Study Design	Population	Sample Size	Key Outcomes
Sieczkowska et al. (2015) [28]	Prospective cohort	Children (3–18 y) on PPI for esophagitis	40	22.5% developed SIBO after 3 months of omeprazole. GI symptoms are more frequent in SIBO-positive cases.
Belei et al. (2017) [29]	Cross-sectional case-control	Obese/overweight vs. lean children	245	SIBO prevalence is 37.6% in obese vs. 3.3% controls. The strong link between SIBO and NAFLD in obese children.
Belei et al. (2018) [30]	RCT	Children with GERD treated with PPI	128	Probiotic + PPI reduced SIBO (6.2% vs. 56.2%). Fewer GI side effects in the probiotic group.
Tapiainen et al. (2019) [31]	Prospective controlled cohort	Newborns (with/without antibiotics)	149	Antibiotic exposure altered microbiota. Dysbiosis persisted despite probiotics. ↑ Antimicrobial resistance genes.
Manzoni et al. (2018) [32]	Secondary analysis of RCT	Preterm VLBW infants	743	Acid-suppressants ↑ LOS risk. Lactoferrin co-admin mitigated risk.
Scott et al. (2016) [33]	Retrospective cohort	UK children (birth to 4 years)	21,714	≥3 antibiotic courses before age 2 ↑ obesity risk at age 4 (OR~1.47).
Todendi et al. (2016) [34]	Cross-sectional	Brazilian children/adolescents	470	Obesity associated with ↑ CRP and IL-6. Central adiposity and CRP gene variant influenced inflammation.
Murugesan et al. (2015) [7]	Cross-sectional	Mexican schoolchildren (5–11 y)	190	Obese children had ↓ butyrate ↑ propionate. Specific microbial shifts, no major dysbiosis.
Guercio Nuzio et al. (2017) [35]	Cross-sectional	Obese children (±NAFLD)	32	↑ Gut permeability, endotoxemia. SIBO only in obese. No mucosal inflammation.
Lai et al. (2019) [36]	RCT	Children (6 mo–6 y) with diarrhea	81	*L. casei* improved symptoms, ↓ inflammatory markers, ↑ and beneficial bacteria.
Korterink et al. (2015) [37]	Cross-sectional	Children (6–18 y) with FGIDs	161	SIBO in 14.3%. Strong link with IBS, symptoms predictive.
Galley et al. (2014) [38]	Cross-sectional	Toddlers born to obese vs. normal-weight mothers	77	Obese mothers → infants with altered microbiota (↑ *Faecalibacterium*, *Blautia*).
McCann et al. (2018) [39]	Cross-sectional (metagenomic)	Infants (1 y) by birth mode	20	Vaginal birth → ↑ virome diversity. Virome clustered by birth mode.
Ignacio et al. (2016) [40]	Cross-sectional	Brazilian children (5–9 y)	51	Obese children had ↑ *Lactobacillus* ↓ *Bifidobacterium*. ↑ *Bacteroides fragilis* group.
Esposito et al. (2020) [41]	Cross-sectional	Obese children with/without SIBO	50	72% had SIBO. ↑ IL-6, TNF-α, IL-8. No correlation with SIBO severity.
Hu et al. (2015) [3]	Cross-sectional	Chinese adolescents (13–17 y)	81	↑ *Prevotella*, ↓ *Bacteroides* in obese. Microbial changes linked to inflammation.
Abdelmalek et al. (2010) [42]	Cross-sectional	Adults with NAFLD	341	Daily fructose intake linked to ↑ fibrosis and inflammation in NAFLD.
Abu-Shanab et al. (2011) [43]	Case-control	Adults with NASH vs. controls	34	SIBO 77.8% in NASH vs. 31.3% controls. ↑ IL-8, TLR-4. Suggests microbial role in NASH.
Boursier et al. (2016) [44]	Cross-sectional	Adults with NAFLD	57	↑ *Bacteroides*, ↓ *Prevotella* in NASH. ↓ *Ruminococcus* in fibrosis. Microbiota predicts NAFLD severity.
Revaiah et al. (2018) [45]	Cross-sectional	Adults on PPIs	147	SIBO: 13.2% on PPI vs. 1.8% with prokinetic. Linked to slower gut transit.
Browning et al. (2011) [46]	Randomized intervention trial	Adults with NAFLD	18	2-week low-carb vs. calorie restriction. Both ↓ liver fat and carb-restricted diets led to greater ↓ (55% vs. 28%). Linked to ↑ketones, fat oxidation.
Van Rossum et al. (2024) [47]	Randomized clinical trial (PRIMAL study)	Preterm infants (28–32 weeks GA)	618	Multistrain probiotics (*B. infantis*, *BB-12*, *L. acidophilus*) did not reduce MDRO+ colonization by day 30 but improved eubiosis scores, aligning the microbiome with that of healthy term infants.
Atazadegan et al. (2023) [48]	RCT	Overweight/obese children (8–18 y)	60	Synbiotic supplementation (*L. coagulans*, *L. indicus* + FOS) significantly reduced waist-to-height ratio but not other body metrics.
Rahkola et al. (2023) [49]	RCT	Preterm neonates (25–35 weeks GA)	68	Direct LGG + Bb12 supplementation significantly increased *B. animalis* and *Lactobacillales* in preterms, while maternal delivery was less effective. Early direct use shapes microbiota composition.

ALT—Alanine Aminotransferase; AST—Aspartate Aminotransferase; BB-12—*Bifidobacterium animalis* subsp. *lactis* BB-12; CRP—C-Reactive Protein; *E. coli*—*Escherichia coli*; FGIDs—Functional Gastrointestinal Disorders; FOS—Fructooligosaccharides; GA—Gestational Age; GERD—Gastroesophageal Reflux Disease; GI—Gastrointestinal; IL—Interleukin; LGG—*Lactobacillus rhamnosus* GG; LOS—Late-Onset Sepsis; MDRO—Multidrug-Resistant Organism; NAFLD—Nonalcoholic Fatty Liver Disease; NASH—Nonalcoholic Steatohepatitis; OR—Odds Ratio; PPI—Proton Pump Inhibitor; RCT—Randomized Controlled Trial; SIBO—Small Intestinal Bacterial Overgrowth; TLR-4—Toll-Like Receptor 4; TNF-α—Tumor Necrosis Factor Alpha; VLBW—Very Low Birth Weight; ↑/↓—Increased/Decreased; → determine.

**Table 4:** Summary of Microbiota Composition by Weight Status and Sex in Pediatric Populations.

Comparison	Dominant Taxa	Key Characteristics
Obese Children	*Lactobacillus*, *Prevotella*, *E. coli*, *Clostridium*	↑ Energy harvest, ↑ inflammation, ↓ butyrate (functional dysbiosis)
Lean Children	*Bifidobacterium*, *Bacteroides*	↑ Microbial diversity, ↓ inflammatory markers
Obese Females	↑ *Lactobacillus*, *Bifidobacterium*, *Alistipes*	Higher microbial diversity, influenced by estrogen
Obese Males	↑ *Bilophila*, *Phascolarctobacterium*	Lower diversity, delayed adult-like microbiota maturation
High-Fiber Diet (e.g., rural)	↑ *Bacteroidetes* (e.g., *Prevotella*)	Anti-inflammatory profile, ↑ SCFAs
Western Diet (high-fat)	↑ *Firmicutes*, ↓ *Bacteroidetes*	Pro-inflammatory taxa, metabolic disruption

SCFAs—Short-Chain Fatty Acids; ↑—Increased; ↓—Decreased; *E. coli*—*Escherichia coli*.

**Table 6:** Prevalence of SIBO in PPI-treated patients (pediatric vs. adult studies).

Pediatrics Population Studies PPI Only					
Study	Design and Population	PPI Exposure	SIBO Diagnostic Method	SIBO Prevalence	Key Findings
Sieczkowska et al., 2015 [28]	Prospective cohort; 40 children (3–18 y) with peptic esophagitis	Omeprazole 1 mg/kg/day for 3 months	GHBT	0% → 22.5% after 3 months	3-month PPI therapy induced SIBO in 22.5% of children. SIBO-positive children had more abdominal pain, bloating, and flatulence.
Cares et al., 2017 [99]	Prospective cohort; 83 children (3–17 y)—PPI users (*n* = 56) vs. controls (*n* = 27)	≥6 months on PPI vs. no PPI	GHBT	8.9% (PPI) vs. 3.7% (control); *p* = 0.359	Higher SIBO was found in the PPI group, but it was not statistically significant. No definitive increased risk of SIBO from PPI use.
PPI + Probiotic					
Belei et al., 2018 [30]	RCT; 128 children (1–18 y) with GERD on PPI; 120 controls	12-week PPI therapy + placebo (*n* = 64) vs. PPI + *L. reuteri* (*n* = 64)	GHBT before/after treatment	PPI + placebo: 56.2% vs. PPI + probiotic: 6.2%; Control: 5%	Probiotic co-administration reduced SIBO to control levels. Significant protective effect against PPI-induced SIBO (*p* < 0.001).
Adult Population Studies PPI Only vs. PPI + Prokinetic					
Revaiah et al., 2018 [45]	Cross-sectional: 147 patients (>12 y) with GERD/FD on long-term PPI	Group A: PPI only vs. Group B: PPI + prokinetic (levosulpiride 75 mg/day)	GHBT	PPI only: 13.2% vs. PPI + prokinetic: 1.8%; *p* = 0.018	Adding a prokinetic significantly reduced SIBO risk compared to PPI alone. Suggests the benefit of motility agents in SIBO prevention.

GERD—Gastroesophageal reflux disease; FD—Functional dyspepsia; GHBT—Glucose hydrogen breath test; PPI—Proton-pump inhibitors; SIBO—Small intestinal bacterial overgrowth.

**Table 7:** Comparison of Glucose and Lactulose Hydrogen Breath Tests for SIBO Diagnosis.

Feature	GHBT	LHBT
Substrate	Glucose	Lactulose
Site of Action	Proximal small intestine	Whole gut
Sensitivity	62–91%	~63.6%
Specificity	75–100%	~67.7%
False Positives	Low	Higher (especially with fast transit)
False Negatives	May miss distal SIBO	Less common

GHBT—Glucose Hydrogen Breath Test; LHBT—Lactulose Hydrogen Breath Test; SIBO—Small Intestinal Bacterial Overgrowth.
